# Supplementary material for: A VSV-based assay quantifies coronavirus Mpro/3CLpro/Nsp5 main protease activity and chemical inhibition
Source: Commun Biol. 2022 Apr 27;5:391. doi: 10.1038/s42003-022-03277-0 (PMC9046202; doi:10.1038/s42003-022-03277-0)
Supplement: Supplementary file 2 — Supplemental Information [file 42003_2022_3277_MOESM2_ESM.pdf]

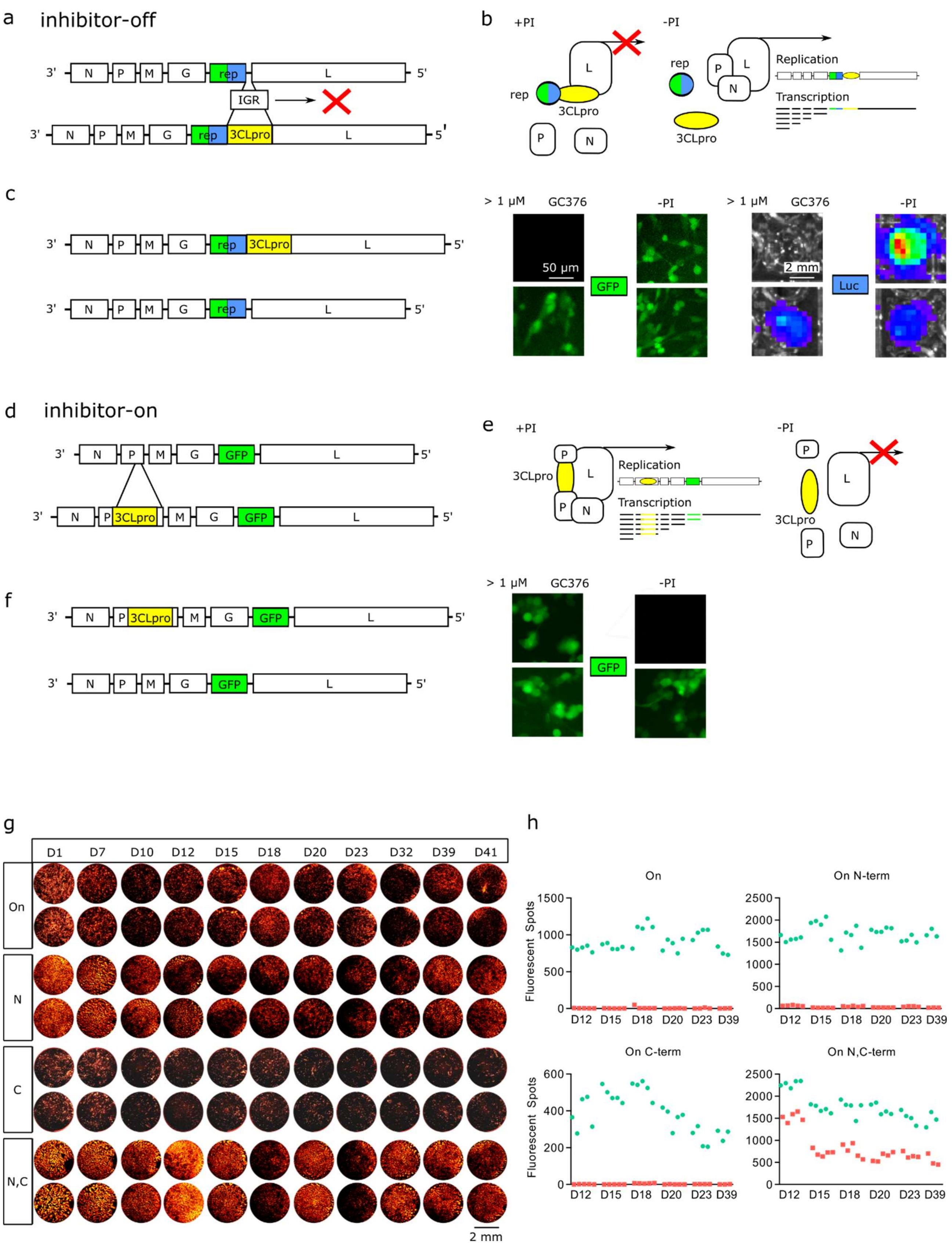

Supplementary Figure 1: Proof of principle of SARS-CoV-2 virus 3C-like protease incorporation into VSV genomes. **a**: Genome scheme of VSV vector with nucleoprotein (N), phosphoprotein (P), matrix protein (M), glycoprotein (G), reporter (rep) and polymerase / large protein (L). Replacement of the intergenic region (IGR) between the reporters GFP or luciferase (rep) and L with insertion of SARS-CoV-2 3C-like protease. **b**: Principle of 3CLpro-Off construct. The SARS-CoV-2 protease releases L in the absence of an inhibitor. The VSV replication and transcription complexes form, producing a reporter gene (rep). Adding a protease inhibitor

stalls L. **c**: Viral reporter genes GFP and *firefly* luciferase expression is blocked in the presence of more than 1 µM of the bona fide protease inhibitor GC376. Control vectors cannot be regulated by addition of GC376. **d**: Genome scheme of wild-type VSV vector and VSV with intramolecular insertion of the SARS-CoV-2 protease into P. **e**: Principle of 3CLpro-On construct. The SARS-CoV-2 protease disintegrates P in the absence of a protease inhibitor. By adding a protease inhibitor, the construct is stabilized and the viral replication and transcription complexes can be formed. **f**: Viral reporter gene GFP expression is facilitated in the presence of more than 1 µM of the bona fide protease inhibitor GC376. Control vectors cannot be regulated by addition of GC376. **g.** Fluorescent signal stability over continuous passaging. BHK21 3CLpro-On expressing cells and its mutated constructs red fluorescent signal in the presence of protease inhibitors (2 independent wells were imaged from each passage, images were enhanced linearly in contrast and brightness to visualize spot signal at low magnification). **h:** Spot count data for selected read-out days for all four 3CLpro-On expressing cell lines over the course of one month. Multiple data points were collected the same day (day 12 to 39) and separated to highlight them. Positive and negative controls are colored in green and red, respectively.

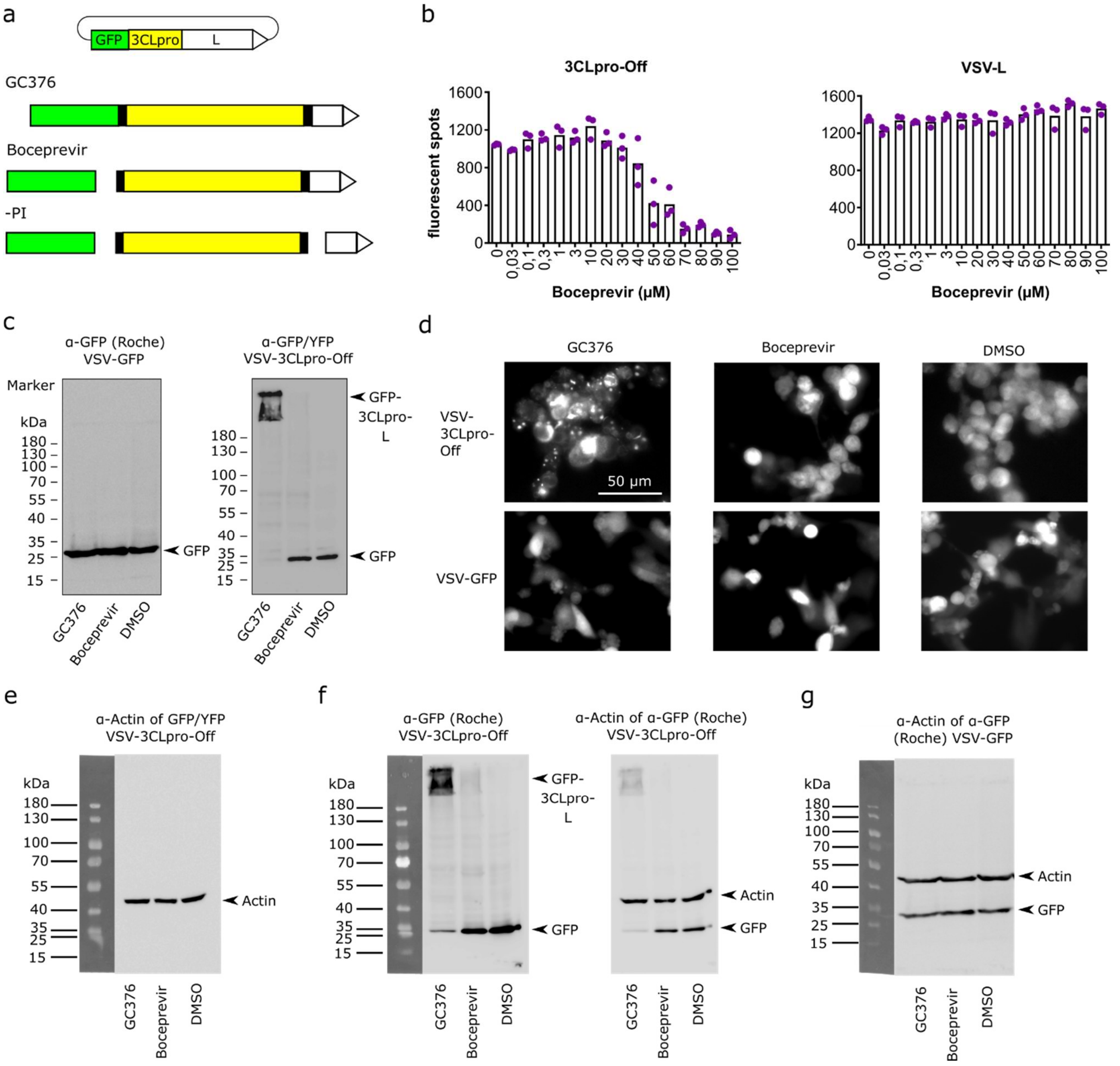

Supplementary Figure 2: a: Schemes of 3CLpro-Off in response to different inhibitors. **b:** 3CLpro-Off and control construct expressing VSV-L in response to different Boceprevir concentrations (n=3 biologically independent replicates per condition). **c:** Anti-GFP immunoblots of cells infected with VSV expressing GFP or VSV expressing 3CLpro-Off treated with either 50 µM GC376, 100 µM Boceprevir or DMSO. **d:** Fluorescence images of cells infected with VSV-3CLpro-Off with 50 µM GC376 or 100 µM Boceprevir. **e:** Mouse α-Actin blot of unstripped rabbit GFP/YFP blot of cell lysates of cells treated with either 50 µM GC376, 100 µM Boceprevir or DMSO. **f:** Blot with mouse α-GFP antibody from Roche and corresponding unstripped mouse α-Actin blot loaded with cell lysates from cells treated with either 50 µM GC376, 100 µM Boceprevir or DMSO. **g:** Mouse α-Actin blot of mouse α-GFP antibody from Roche VSV-GFP blot loaded with cell lysates from cells treated with either 50 µM GC376, 100 µM Boceprevir or DMSO.

Supplemental table 1: Additional protease sequences tested in this project

**Rousettus bat coronavirus HKU9** (NCBI Reference Sequence: NC_009021.1)
SVASAALQAGLTRMAHPSGLVEPCLVKVNYGSMTLNGIWLDNFVICPRHVMCSRDELANPDYPRLSMRAANYDFHVS<br>QNGHNIRVIGHTMEGSLLKLTVDVNNPKTPAYSFIRVSTGQAMSLLACYDGLPTGVYTCTLRSNGTMRASFLCGSCGSP<br>GFVMNGKEVQFCYLHQLELPNGTHTGTDFSGVFYGPFEDKQVPQLAAPDCTITVNVLAWLYAAVLSGENWFLTKSSISP<br>AEFNNCAVKYMCQSVTSESLQVLQPLAAKTGISVERMLSALKVLLSAGFCGRTIMGSCSLEDEHTPYDIGRQMLGVKLQ<br>GKFQSMFR
**Human coronavirus 229E (**NCBI Reference Sequence: KF293666.1)
VSYGSTLQAGLRKMAQPSGFVEKCVVRVCYGNTVLNGLWLGDIVYCPRHVIASNTTSAIDYDHEYSIMRLHNFSIISGTA<br>FLGVVGATMHGVTLKIKVSQTNMHTPRHSFRTLKSGEGFNILACYDGCAQGVFGVNMRTNWTIRGSFINGACGSPGY<br>NLKNGEVEFVYMHQIELGSGSHVGSSFDGVMYGGFEDQPNLQVESANQMLTVNVVAFLYAAILNGCTWWLKGEKLF<br>VEHYNEWAQANGFTAMNGEDAFSILAAKTGVCVERLLHAIQVLNNGFGGKQILGYSSLNDEFSINEVVKQMFGVNLQS<br>GKTTSMF
**Human coronavirus NL63** (NCBI Reference Sequence: YP_010229075.1)
ISYNSTLQSGLKKMAQPSGCVERCVVRVCYGSTVLNGVWLGDTVTCPRHVIAPSTTVLIDYDHAYSTMRLHNFSVSHNG<br>VFLGVVGVTMHGSVLRIKVSQSNVHTPKHVFKTLKPGDSFNILACYEGIASGVFGVNLRTNFTIKGSFINGACGSPGYNV<br>RNDGTVEFCYLHQIELGSGAHVGSDFTGSVYGNFDDQPSLQVESANLMLSDNVVAFLYAALLNGCRWWLCSTRVNVD<br>GFNEWAMANGYTSVSSVECYSILAAKTGVSVEQLLASIQHLHEGFGGKNILGYSSLCDEFTLAEVVKQMYGVNLQSGKVI<br>FGL
**Middle East respiratory syndrome-related coronavirus (MERS)** (NCBI Reference Sequence: KF600615.1)
SITSGVLQSGLVKMSHPSGDVEACMVQVTCGSMTLNGLWLDNTVWCPRHVMCPADQLSDPNYDALLISMTNHSFSV<br>QKHIGAPANLRVVGHAMQGTLLKLTVDVANPSTPAYTFTTVKPGAAFSVLACYNGRPTGTFTVVMRPNYTIKGSFLCGS<br>CGSVGYTKEGSVINFCYMHQMELANGTHTGSAFDGTMYGAFMDKQVHQVQLTDKYCSVNVVAWLYAAILNGCAWF<br>VKPNRTSVVSFNEWALANQFTEFVGTQSVDMLAVKTGVAIEQLLYAIQQLYTGFQGKQILGSTMLEDEFTPEDVNMQI<br>MGVVMQSGVRKVTY
**Mouse Hepatitis Virus (MHV)** (NCBI Reference Sequence: NP_045298.1)
SVTTSFLQSGIVKMVSPTSKVEPCIVSVTYGNMTLNGLWLDDKVYCPRHVICSSADMTDPDYPNLLCRVTSSDFCVMSG<br>RMSLTVMSYQMQGCQLVLTVTLQNPNTPKYSFGVVKPGETFTVLAAYNGRPQGAFHVTLRSSHTIKGSFLCGSCGSVG<br>YVLTGDSVRFVYMHQLELSTGCHTGTDFSGNFYGPYRDAQVVQLPVQDYTQTVNVVAWLYAAIFNRCNWFVQSDSCS<br>LEEFNVWAMTNGFSSIKADLVLDALASMTGVTVEQVLAAIKRLHSGFQGKQILGSCVLEDETPSDVYQQLAGVKLQSKR<br>TRVIK
**Poliovirus** (NCBI Reference Sequence: ACH87554.1)
TIRTAKVQGPGFDYAVAMAKRNIVTATTSKGEFTMLGVHDNVAILPTHASPGESIVIDGKEVEILDAKALEDQAGTNLEIT<br>IITLKRNEKFRDIRPHIPTQITETNDGVLIVNTSKYPNMYVPVGAVTEQGYLNLGGRQTARTLMYNFPTRAGQCGGVITCT<br>GKVIGMHVGGNGSHGFAAALKRSYFTQSQGEIQWMRP
**SARS-CoV-1** (NCBI Reference Sequence: AAP82976.1)
SITSAVLQSGFRKMAFPSGKVEGCMVQVTCGTTTLNGLWLDDTVYCPRHVICTAEDMLNPNYEDLLIRKSNHSFLVQAG<br>NVQLRVIGHSMQNCLLRLKVDTSNPKTPKYKFVRIQPGQTFSVLACYNGSPSGVYQCAMRPNHTIKGSFLNGSCGSVGF<br>NIDYDCVSFCYMHHMELPTGVHAGTDLEGKFYGPFVDRQTAQAAGTDTTITLNVLAWLYAAVINGDRWFLNRFTTTLN<br>DFNLVAMKYNYEPLTQDHVDILGPLSAQTGIAVLDMCAALKELLQNGMNGRTILGSTILEDEFTPFDVVRQCSGVTFQG<br>KFKKIVK
**SARS-CoV-2** (NCBI Reference Sequence: NC_045512.2)
ITSAVLQSGFRKMAFPSGKVEGCMVQVTCGTTTLNGLWLDDVVYCPRHVICTSEDMLNPNYEDLLIRKSNHNFLVQAG<br>NVQLRVIGHSMQNCVLKLKVDTANPKTPKYKFVRIQPGQTFSVLACYNGSPSGVYQCAMRPNFTIKGSFLNGSCGSVGF<br>NIDYDCVSFCYMHHMELPTGVHAGTDLEGNFYGPFVDRQTAQAAGTDTTITVNVLAWLYAAVINGDRWFLNRFTTTL<br>NDFNLVAMKYNYEPLTQDHVDILGPLSAQTGIAVLDMCASLKELLQNGMNGRTILGSALLEDEFTPFDVVRQCSGVTFQ<br>SAVKRTI

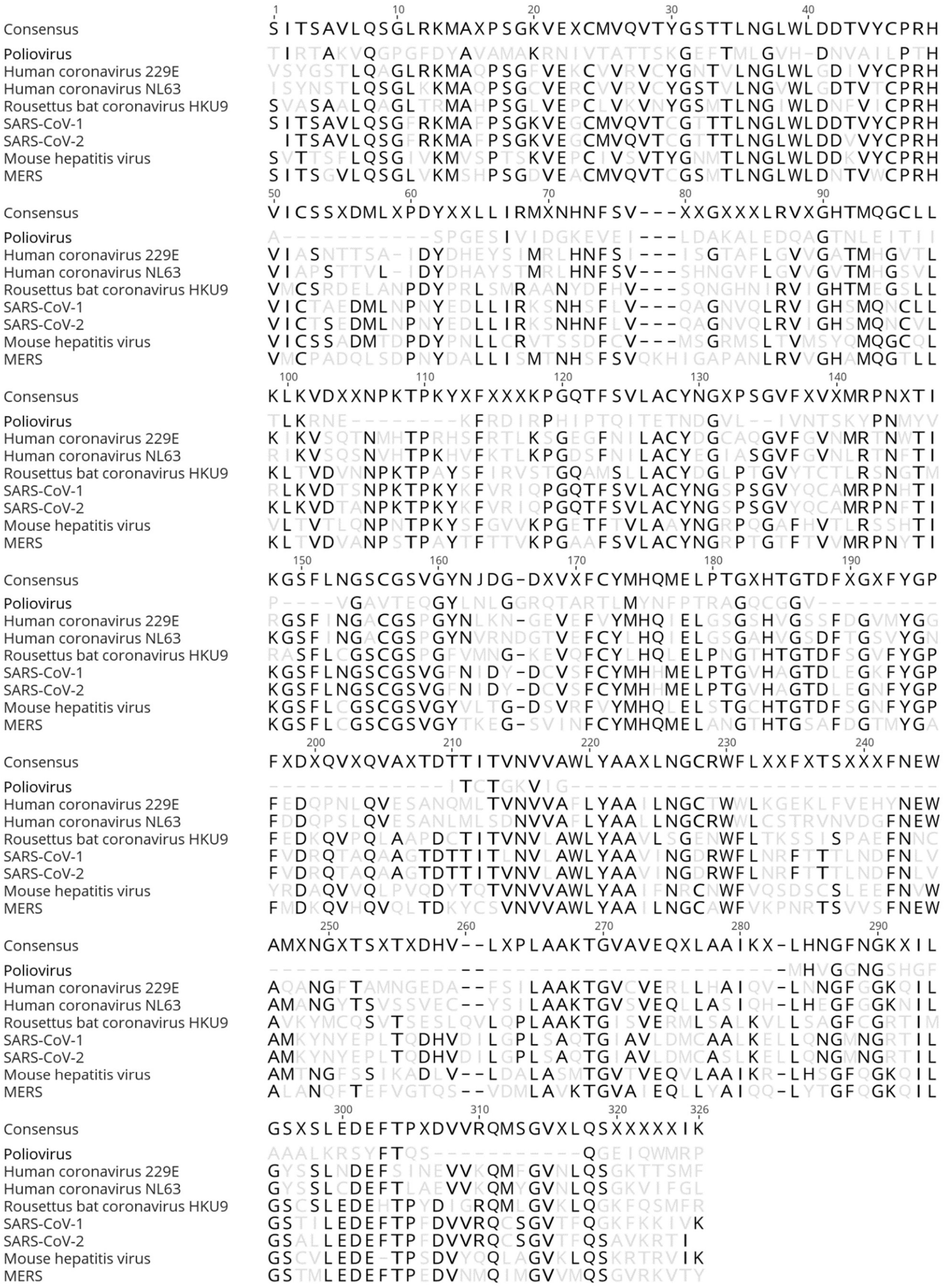

Supplementary Figure 3: Amino acid sequence alignment of main proteases of Poliovirus, Human coronaviruses 229E and NL63, Rousettus bat coronavirus HKU9, SARS-Cov-1 and -2, mouse hepatitis virus and MERS.

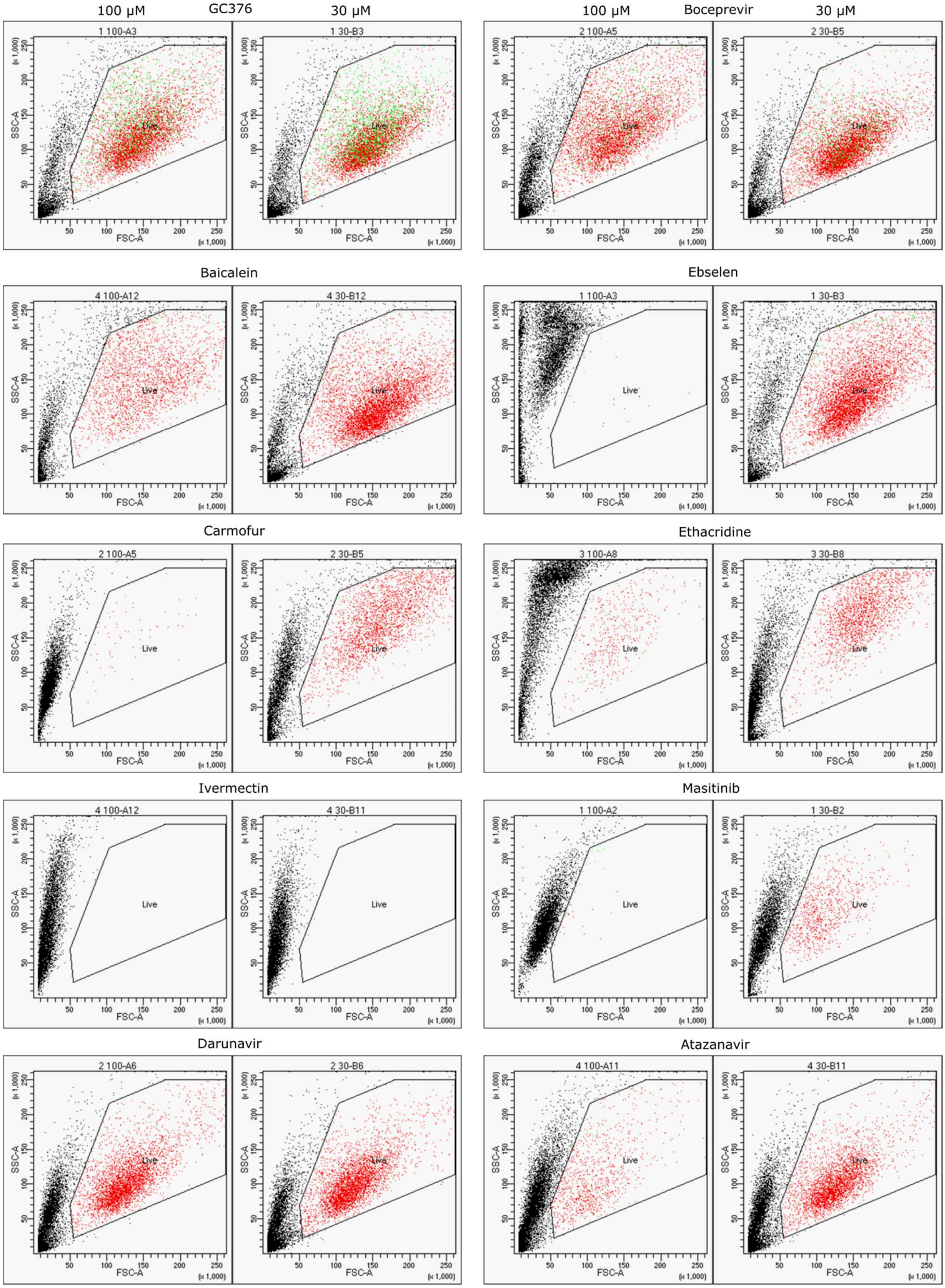

Supplementary Figure 4: Gating on live cells treated with different compounds and infected with VSV-ΔP-dsRed. One representative replicate of 100 and 30 µM samples are shown. Red dots indicate below cut-off for following dsRed gate. Green dots indicate dsRed positivity in following gate (see also **Figure S5 and 6**).

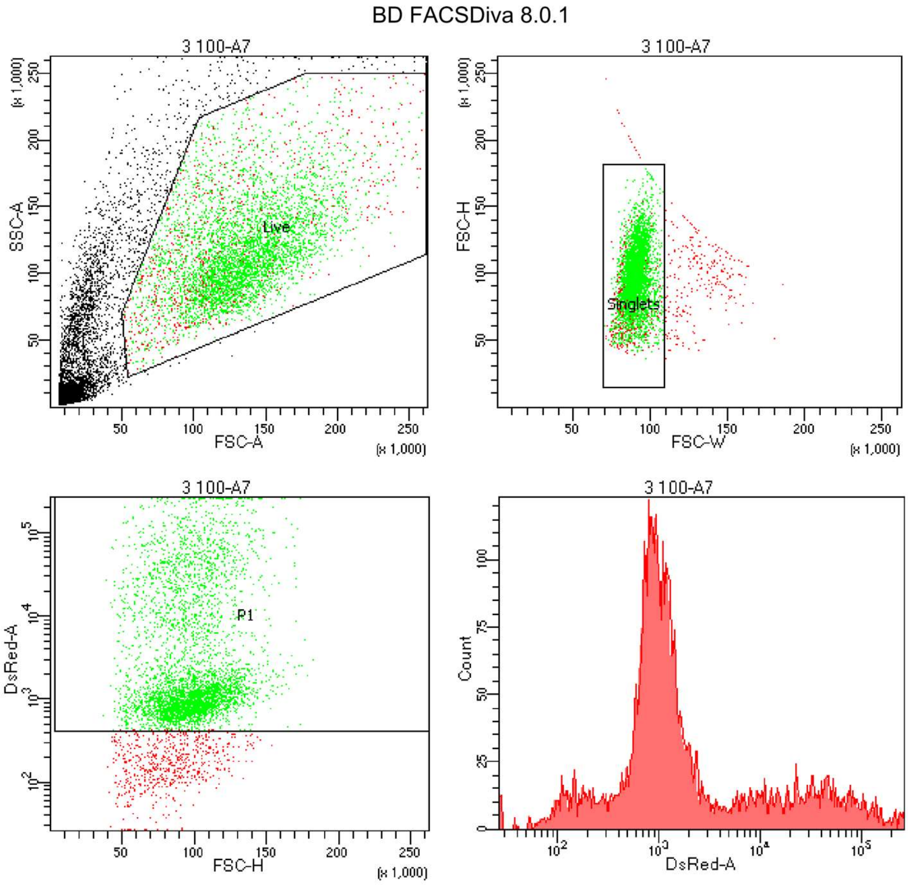

Well: A7

Population	#Events	%Parent	%Total
All Events	10,000	####	100.0
Live	5,240	52.4	52.4
Singlets	4,994	95.3	49.9
P1	4,362	87.3	43.6

Experiment Name:	Mpro cis-cleavage assay-...
Specimen Name:	3 100
Well Name:	A7
Record Date:	Dec 5, 2021 10:47:28 PM
$OP:	Administrator
GUID:	902c16f6-69ec-4a8e-974...

Population	#Events	%Parent	FSC-H Mean	DsRed-A Mean
Singlets	4,994	95.3	95,493	15,136
P1	4,362	87.3	97,437	17,301

Supplementary Figure 5: Exemplary FACS read-out and gating strategy of VSV-ΔP-dsRed infected BHK-21 cells treated with 100 µM PF-07321332. DsRed-A Mean and P1 Events were used for quantitative read-outs. Red dots indicate below cut-off in dsRed gate. Green dots indicate dsRed positive cells.

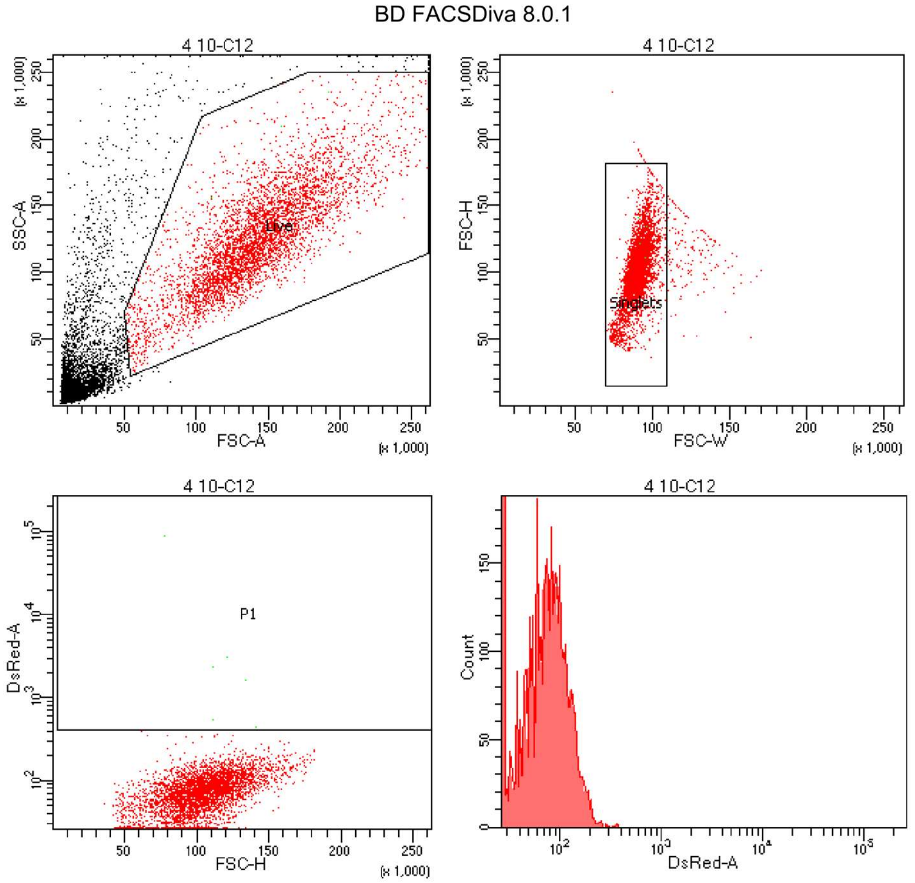

Well: C12

Population	#Events	%Parent	%Total
All Events	10,000	####	100.0
Live	4,822	48.2	48.2
Singlets	4,655	96.5	46.6
P1	6	0.1	0.1

Experiment Name:	Mpro cis-cleavage assay-...
Specimen Name:	4 10
Well Name:	C12
Record Date:	Dec 6, 2021 3:32:59 PM
$OP:	Administrator
GUID:	2e63cb57-a243-4453-bc...

Population	#Events	%Parent	FSC-H Mean	DsRed-A Mean
Singlets	4,655	96.5	101,835	98
P1	6	0.1	115,475	16,224

Supplementary Figure 6: Exemplary FACS read-out and gating strategy of VSV-ΔP-dsRed infected, but untreated BHK-21 cells. DsRed-A Mean and P1 Events were used for quantitative read-outs. Red dots indicate below cut-off in dsRed gate. Green dots indicate dsRed positive cells.

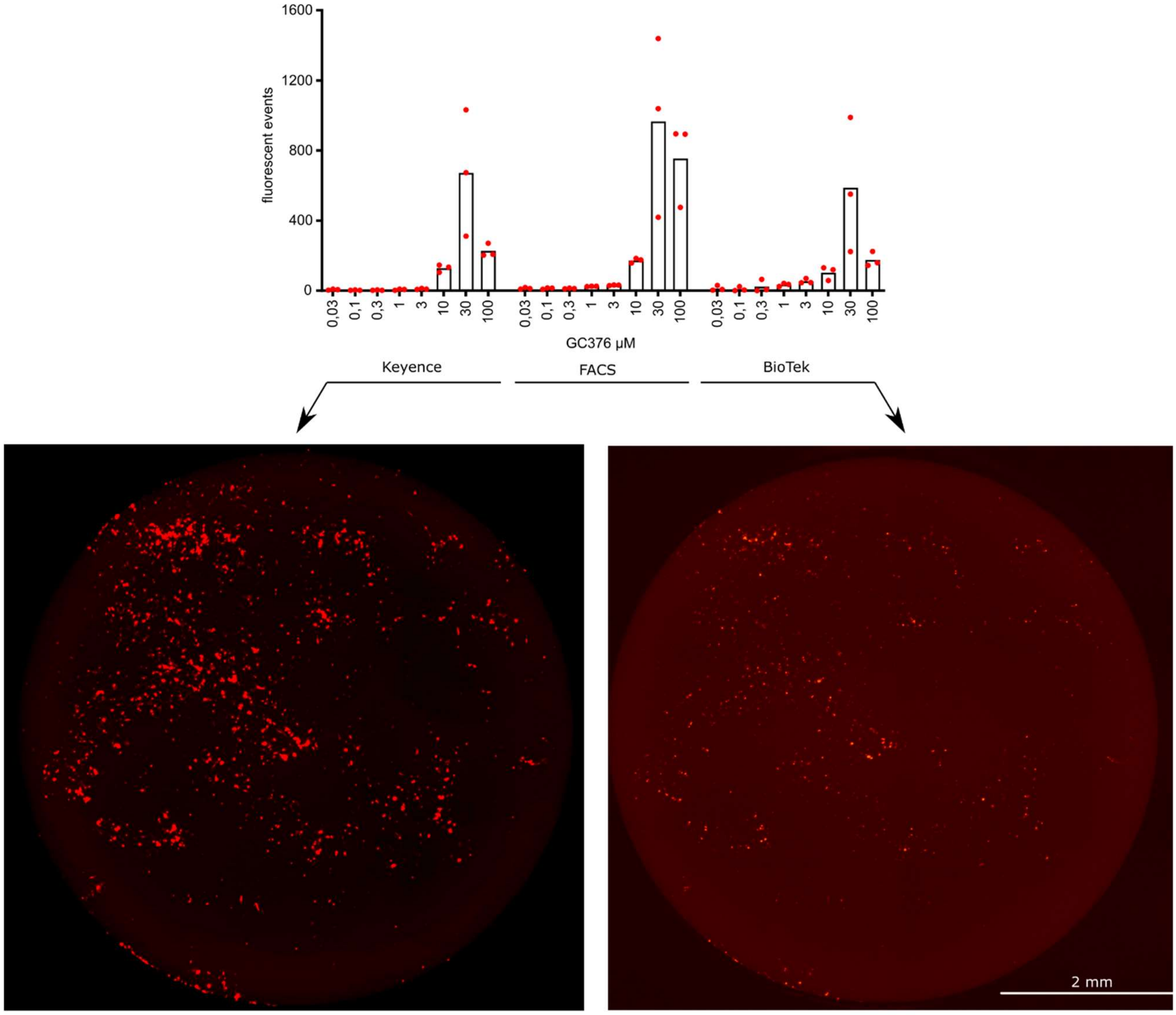

Supplementary Figure 7: Comparison of different fluorescent-based read-out methods with Rousettus bat coronavirus HKU9 main protease gain-of-signal BHK-21 cells (n=3 biologically independent replicates per condition). Representative images of the same 30 µM GC376 well was chosen for comparison of camera sensitivity between Keyence and BioTek readers.

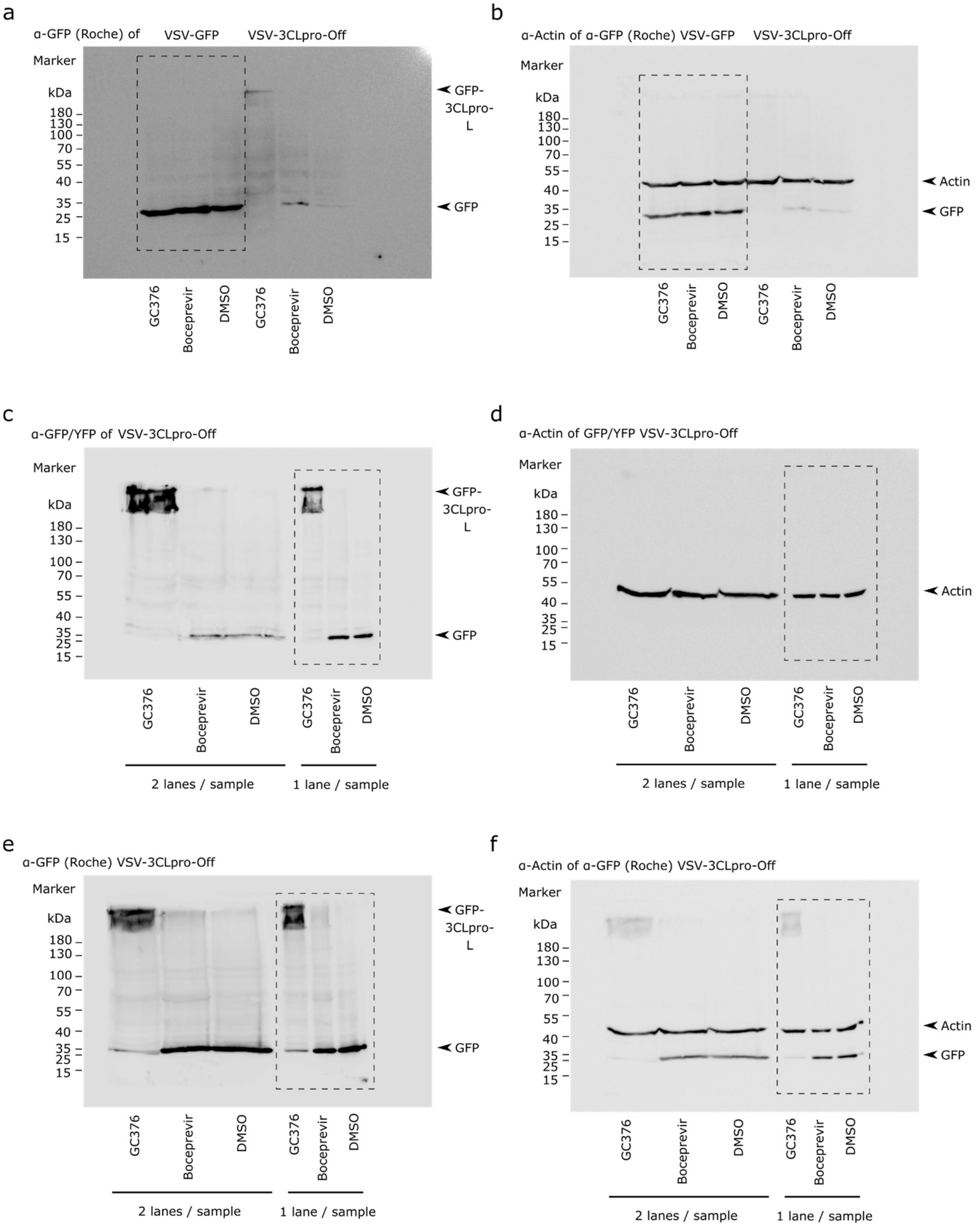

Supplementary Figure 8: a: Mouse anti-GFP (antibody from Roche) immunoblot of cells infected with VSV expressing GFP or VSV expressing 3CLpro-Off treated with either 50 µM GC376, 100 µM Boceprevir or DMSO. **b:** Unstripped mouse anti-actin blot of mouse anti-GFP immunoblot in panel a. **c:** Rabbit anti-GFP/YFP immunoblot of cells infected with VSV expressing 3CLpro-Off treated with either 50 µM GC376, 100 µM Boceprevir or DMSO. In the left side of the blot, 2 lanes were joined for each sample to load a larger sample volume. **d:** Unstripped mouse anti-actin blot of rabbit anti-GFP/YFP immunoblot in panel c. **e:** Mouse anti-GFP (antibody from Roche) immunoblot of cells infected with VSV expressing 3CLpro-Off treated with either

50 µM GC376, 100 µM Boceprevir or DMSO. In the left side of the blot, 2 lanes were joined for each sample to load a larger sample volume. **f:** Unstripped mouse anti-actin blot of mouse anti-GFP (Roche) immunoblot in panel e. Dashed rectangles indicate which part of the blots was used for **Supplementary Figure 2**.
