# Supplementary material for: A VSV-based assay quantifies coronavirus Mpro/3CLpro/Nsp5 main protease activity and chemical inhibition
Source: Commun Biol. 2022 Apr 27;5:391. doi: 10.1038/s42003-022-03277-0 (PMC9046202; doi:10.1038/s42003-022-03277-0)
Supplement: Supplementary file 3 — Description of Additional Supplementary Files [file 42003_2022_3277_MOESM3_ESM.pdf]

Description of Additional Supplementary Files

File name: Supplementary Data 1
Description: Data used to generate graphs of main figures.
