# Supplementary material for: A VSV-based assay quantifies coronavirus Mpro/3CLpro/Nsp5 main protease activity and chemical inhibition
Source: Commun Biol. 2022 Apr 27;5:391. doi: 10.1038/s42003-022-03277-0 (PMC9046202; doi:10.1038/s42003-022-03277-0)
Supplement: Supplementary file 5 — Reporting Summary [file 42003_2022_3277_MOESM5_ESM.pdf]

nature portfolio

Corresponding author(s): Emmanuel Heilmann

Last updated by author(s): 2022.02.28

Reporting Summary

Nature Portfolio wishes to improve the reproducibility of the work that we publish. This form provides structure for consistency and transparency in reporting. For further information on Nature Portfolio policies, see our Editorial Policies and the Editorial Policy Checklist.

Statistics

For all statistical analyses, confirm that the following items are present in the figure legend, table legend, main text, or Methods section.

n/a	Confirmed	
☐	☒	The exact sample size (n) for each experimental group/condition, given as a discrete number and unit of measurement
☐	☒	A statement on whether measurements were taken from distinct samples or whether the same sample was measured repeatedly
☒	☐	The statistical test(s) used AND whether they are one- or two-sided<br>*Only common tests should be described solely by name; describe more complex techniques in the Methods section.*
☒	☐	A description of all covariates tested
☒	☐	A description of any assumptions or corrections, such as tests of normality and adjustment for multiple comparisons
☒	☐	A full description of the statistical parameters including central tendency (e.g. means) or other basic estimates (e.g. regression coefficient) AND variation (e.g. standard deviation) or associated estimates of uncertainty (e.g. confidence intervals)
☒	☐	For null hypothesis testing, the test statistic (e.g. F, t, r) with confidence intervals, effect sizes, degrees of freedom and P value noted<br>*Give P values as exact values whenever suitable.*
☒	☐	For Bayesian analysis, information on the choice of priors and Markov chain Monte Carlo settings
☒	☐	For hierarchical and complex designs, identification of the appropriate level for tests and full reporting of outcomes
☒	☐	Estimates of effect sizes (e.g. Cohen's d, Pearson's r), indicating how they were calculated

Our web collection on statistics for biologists contains articles on many of the points above.

Software and code

Policy information about availability of computer code

Data collection	Automated fluorescent spot counting: Fluoro/ImmunoSpot CTL switchboard 2.7.2<br>Luciferase imaging: Caliper Live Sciences-Living Image® Software 4.3.1.<br>Microscopy: NIS-Elements BR 4.20.01, VisiView software (Visitron) 4.1.0.3.
Data analysis	Automated fluorescent spot counting and quality control: Fluoro/ImmunoSpot CTL switchboard 2.7.2<br>Generation of graphs: GraphPad Prism 8<br>Sequence analysis: Geneious Prime 2019.2.1<br>Luciferase quantification: Caliper Live Sciences-Living Image® Software 4.3.1.<br>Figure generation and processing: Inkscape 0.92.3

For manuscripts utilizing custom algorithms or software that are central to the research but not yet described in published literature, software must be made available to editors and reviewers. We strongly encourage code deposition in a community repository (e.g. GitHub). See the Nature Portfolio guidelines for submitting code & software for further information.

Data

Policy information about availability of data

All manuscripts must include a data availability statement. This statement should provide the following information, where applicable:

- Accession codes, unique identifiers, or web links for publicly available datasets
- A description of any restrictions on data availability
- For clinical datasets or third party data, please ensure that the statement adheres to our policy

All pertinent data to support this study are included in the manuscript and supplementary files. If required, further data supporting the findings are available upon request.

Field-specific reporting

Please select the one below that is the best fit for your research. If you are not sure, read the appropriate sections before making your selection.

☒ Life sciences ☐ Behavioural & social sciences ☐ Ecological, evolutionary & environmental sciences

For a reference copy of the document with all sections, see nature.com/documents/nr-reporting-summary-flat.pdf

Life sciences study design

All studies must disclose on these points even when the disclosure is negative.

Sample size	Sample sizes were chosen empirically based on experience from our previous studies.
Data exclusions	Automated fluorescent spot counting and quality control with the Fluoro/ImmunoSpot CTL switchboard 2.7.2 software includes automated fiber removal as well as manual control, which is mostly performed to exclude fibers that were not recognized by the software.
Replication	Dose response curves had 3 or more biological replicates per condition and were repeated with different concentration ranges to find a demonstrative range. Western blots were repeated twice and performed with two different GFP antibodies (Roche anti GFP; anti GFP/YFP.)
Randomization	Randomization was not applicable in this study, because there were no study participants needed for experimental findings.
Blinding	For most parts, investigators were not blinded during data collection or analysis because planning, execution, and analysis of the studies was performed by the same personnel.

Reporting for specific materials, systems and methods

We require information from authors about some types of materials, experimental systems and methods used in many studies. Here, indicate whether each material, system or method listed is relevant to your study. If you are not sure if a list item applies to your research, read the appropriate section before selecting a response.

Materials & experimental systems

n/a	Involved in the study	
☐	☒	Antibodies
☐	☒	Eukaryotic cell lines
☒	☐	Palaeontology and archaeology
☒	☐	Animals and other organisms
☒	☐	Human research participants
☒	☐	Clinical data
☒	☐	Dual use research of concern

Methods

n/a	Involved in the study	
☒	☐	ChIP-seq
☒	☐	Flow cytometry
☒	☐	MRI-based neuroimaging

Antibodies

Antibodies used	Primary antibody rabbit-α-GFP/YFP produced by Stephan Geley (conc.: 0.3 mg/mL)<br>Primary antibody mouse-α-GFP clones 7.1 and 13.1 (Roche, Basel, Switzerland)<br>Primary antibody mouse-α-β-Actin A2228-200UL (Sigma-Aldrich, St. Louis, USA)<br>Secondary antibody goat-α-rabbit (H+L) peroxidase-conjugated AffiniPure Goat (Jackson, ImmunoResearch, Pannsylvania, USA)<br>Secondary antibody goat-α-mouse (H+L) peroxidase-conjugated AffiniPure Goat (Jackson, ImmunoResearch, Pannsylvania, USA)
Validation	Primary antibody mouse-α-β-Actin A2228-200UL (Sigma-Aldrich, St. Louis, USA):<br>Site-directed MT1-MMP trafficking and surface insertion regulate AChR clustering and remodeling at developing NMJs.<br>Zora Chui-Kuen Chan et al.

eLife, 9 (2020-03-26)

Antioxidant diet, gender and age affect renal expression of nitric oxide synthases in obese diabetic rats.
Yuriy Slyvka et al.
Nitric oxide : biology and chemistry, 24(1), 50-60 (2010-11-26)

The interleukin-33-mediated inhibition of expression of two key genes implicated in atherosclerosis in human macrophages requires MAP kinase, phosphoinositide 3-kinase and nuclear factor-κB signaling pathways.
Melanie L Buckley et al.
Scientific reports, 9(1), 11317-11317 (2019-08-07)

Primary antibody mouse-α-GFP clones 7.1 and 13.1 (Roche, Basel, Switzerland):
DDX3X and specific initiation factors modulate FMR1 repeat-associated non-AUG-initiated translation.
Alexander E Linsalata et al.
EMBO reports, 20(9), e47498-e47498 (2019-07-28)

me101 is a new allele of rad-51.
Baptiste Roelens et al.
microPublication biology, 2019 (2019-04-26)

Hepatic DNAJB9 Drives Anabolic Biasing to Reduce Steatosis and Obesity.
Fangfang Sun et al.
Cell reports, 30(6), 1835-1847 (2020-02-13)

Eukaryotic cell lines

Policy information about cell lines

Cell line source(s)	BHK-21 cells (American Type Culture Collection (ATCC), Manassas, VA),<br>293T cells (ATCC) were used to generate 293T expressing SARS-CoV-2 3CLpro constructs and VSV-P/L
Authentication	All cell lines were checked by morphology compared to cell bank references. Genomic authentication was not performed.
Mycoplasma contamination	All cell lines were tested negative for mycoplasma contamination
Commonly misidentified lines<br>(See ICLAC register)	No commonly misidentified cell lines were used in this study.
